# Genetic trends in body measurements at birth for Arabian horse in Türkiye

**DOI:** 10.5194/aab-67-561-2024

**Published:** 2024-11-28

**Authors:** Özlem Hacan, Mustafa Tekerli, Samet Çinkaya, Mustafa Demirtaş

**Affiliations:** 1 Department of Animal Science, Faculty of Veterinary Medicine, Afyon Kocatepe University, Afyonkarahisar, Türkiye

## Abstract

Changes in genetic variation in body measurements are a subject of interest. This study aimed to understand the changes in the genetic effects of body measurement at birth in Turkish Arabian foals over the years. Furthermore, estimating the sources of variation in body measurements at birth in Turkish Arabian foals, considering additive genetic, maternal genetic, and maternal permanent effects and the covariance between offspring and dams in animal models, was the objective of this study. The records for birth weight (BW), wither height (WH), chest circumference (CC), and cannon-bone circumference (CBC) of 2624 Arabian foals born between 1987 and 2007 in the Anadolu, Karacabey, and Sultansuyu agricultural enterprises were used in the analyses. Variance analysis for non-genetic effects showed that the effects of the farm, year of birth, sex, and dam age were significant ( P<0.001 ) for all traits. Estimation of variance components and genetic parameters for body measurements was performed with the average information restricted maximum likelihood algorithm using six univariate animal models in the WOMBAT software. The best-fit model for each trait was identified based on Akaike's information criterion (AIC). Genetic trends were determined by performing linear regression analysis on the estimated breeding value (EBV) of the animals based on their year of birth. Additive direct heritabilities for BW, WH, CC, and CBC were 0.10 ± 0.04, 0.41 ± 0.07, 0.06 ± 0.03, and 0.30 ± 0.07, respectively. The estimates of maternal heritability for the corresponding traits were 0.24 ± 0.03, 0.05 ± 0.03, 0.09 ± 0.03, and 0.13 ± 0.03, respectively. Additive–maternal genetic correlations for BW, WH, CC, and CBC were 0.33, - 0.13, - 0.19, and - 0.22, respectively. Genetic and phenotypic correlations were analyzed with multivariate animal models considering additive genetic, maternal genetic, and maternal permanent effects and ranged from 0.340 to 0.924. The low to moderate direct and maternal heritabilities with additive–maternal genetic correlations showed that the variation in morphometric traits in foals could be affected by these factors and needs to be considered. Genetic trends showed increased weight and chest circumference in foals at birth. Based on these findings, breeders may consider these traits when selecting horses in future breeding programs.

## 1 Introduction

The horse was domesticated in central Asia in approximately 3000 BCE. Horses have served people for centuries, not only for their meat and milk but also for transportation and agricultural purposes, following the process of domestication. Today, horses are also used for racing, sports, and show purposes and have a significant psychological and social impact on society (Arpacık, 1999). In recent years, hippotherapy has brought new perspectives in the treatment of some psychological disorders and disabled people. Selection can be applied more effectively by identifying the environmental factors that affect birth weight and body size. Body size is the most important indicator of animal growth and development. Each horse breed has specific body measurements in different age periods. Horses that fail to achieve the desired body size during these specific age periods are recognized as exhibiting abnormal development (Arpacık, 1999). Depending on the purpose of the yield and service (drafting, riding, racing), different body characteristics are required (Akçapınar et al., 2005; Yıldırım, 2023). A horse with elevated and elongated withers and well-formed muscles is desirable for speed. It is generally desired that the chest cavity be wide and strong for robust lungs and hearts in horses. The cannon-bone circumference (CBC) is also critical because it represents skeletal structure (Batu, 1951). Studies exploring the additive genetic and maternal influences on body size of Arabian foals are scarce. The study of Duru et al. (2017) is focused on the genetic parameters of body measurements for Arabian horses aged 1 or 2 years. Çilek (2012) investigated the variance components of these traits in foals. However, there is a lack of studies on maternal effects and genetic trends in body measurements of Arabian foals. In Türkiye, Arabian horses are raised purely for race performance by the state on Karacabey, Anadolu, and Sultansuyu stud farms. The genetic effects of selecting for performance on body measurements at birth in horses raised on Karacabey, Anadolu, and Sultansuyu stud farms remain poorly understood. Therefore, our study aimed to investigate the changes in genetic variation in these traits. Another primary objective of our study was to examine the additive and maternal effects on the variation observed in body measurements of Arabian foals at birth.

## 2 Material and methods

The material for this study comprised records from foals maintained at the Anadolu, Karacabey, and Sultansuyu agricultural enterprises of the TİGEM between 1987 and 2007. Body weight, wither height, chest circumference, and cannon-bone circumference of 2624 foals were used in the analyses. The data used in the study were obtained from each enterprise, courtesy of TİGEM. The least-squares analysis aiming to reveal the effects of the environmental factors on birth weight (BW), wither height (WH), chest circumference (CC), and CBC was performed using Minitab 18 with the following model:

$$Y_{ijklmn}=\mu +F_{i}+{\text{BY}}_{j}+{\text{BM}}_{k}+S_{l}+{\text{DA}}_{m}+e_{ijklmn},$$

where $Y_{ijklmn}$ is a body measurement related to the trait for observation n , for farm operation i ( i = Karacabey, Anadolu, and Sultansuyu), birth year j ( j = 1987, 1988, …, 2007), birth month k ( k = 1, 2, …, 5), sex l ( l = male, female), and dam age m ( m = ≤ 7, ≥ 8 and ≤ 12, and ≥ 13); μ is the overall mean; F is the effect of farm; BY is the effect of birth year; BM is the effect of birth month; S is the effect of sex; DA is the effect of dam age; and e is the random residual N (0, $\sigma^{2}$ ). Multiple comparisons were performed using Tukey's test.

The average information restricted maximum likelihood (AI-REML) method was used to estimate the variance and covariance components for body measurements of foals at birth using the WOMBAT (Meyer, 2007). Six univariate animal models with different combinations of additive genetic, maternal genetic, and maternal permanent environmental effects with or without covariance between offspring and dams were used to estimate genetic parameters for each trait. Non-genetic environmental factors were included in the models. The statistical models are as follows:

1Y=Xβ+Zaa+e2Y=Xβ+Zaa+Zcc+e3Y=Xβ+Zaa+Zmm+eCov(a,m)=04Y=Xβ+Zaa+Zmm+eCov(a,m)=Aσam5Y=Xβ+Zaa+Zmm+Zcc+eCov(a,m)=06Y=Xβ+Zaa+Zmm+Zcc+eCov(a,m)=Aσam,

where Y is the vector related to data. In the models, fixed, additive, genetic, maternal additive, maternal permanent environmental, and residual effects are indicated as vectors β , a , m , c , and e , respectively, and their corresponding incidence matrices are, X , $Z_{a}$ , $Z_{m}$ , and $Z_{c}$ , respectively. Maternal additive genetic variance is denoted as $\sigma_{m}^{2}$ , and direct additive genetic variance is denoted as $\sigma_{a}^{2}$ . The maternal permanent environmental and residual variances are represented as $\sigma_{\text{pe}}^{2}$ and $\sigma_{e}^{2}$ , while $\sigma_{\text{am}}$ stands for the additive–maternal covariance. Identity matrices with orders equal to the number of dams and the number of lambs are denoted as $I_{d}$ and $I_{n}$ , respectively. A represents the numerator relationship matrix between the animals.

The determination of the most fitted model for each trait considering Akaike's information criterion (AIC) was used with the log-likelihood ratio test. The formula for AIC is as follows (Akaike, 1973):

$$\mathrm{AIC}=-2\text{log}L_{i}+2p_{i},$$

where $\text{log}L_{i}$ is the maximized log likelihood of model i at convergence, and $p_{i}$ is the number of random (co)variance parameters of model i . The model with the smallest AIC value was the most suitable.

The total heritability ( $H_{T}^{2}$ ) calculation for each model was performed with the equation $h_{T}^{2}=(\sigma_{a}^{2}+0.5\sigma_{m}^{2}+1.5\sigma_{\text{am}})/\sigma_{P}^{2}$ (Willham, 1972). The multivariate animal model with additive genetic, maternal genetic, and maternal permanent effects in WOMBAT, incorporating fixed effects, was applied in the estimation of genetic and phenotypic correlations between body measurements.

The estimated breeding values (EBVs) were obtained using the best-fit model of a trait. The annual mean EBVs were calculated by averaging the EBVs of foals born each year. Genetic trends for each body measurement trait were estimated by performing linear regression analysis using Minitab, where a regression was calculated between the yearly mean EBVs and the corresponding year of birth.

**Table 1:** Characteristics of the data structure for body measurement at birth in Turkish Arabian foals.

Item	Traits			
	BW	WH	CC	CBC
Number of records	2607	2607	2592	2615
Number of dams	1164	1163	1164	1164
Average number of progenies per dam	2.24	2.24	2.23	2.25
Mean	45.46	96.98	80.59	11.03
Standard deviation	3.74	3.89	2.98	0.92
Min	34.00	84.00	71.00	9.00
Max	57.00	109.00	90.00	13.50
Average inbreeding coefficient (%)	3.04	3.04	3.03	3.04

## 3 Results

Table 1 provides an overview of the data structure, detailing body measurements collected at birth in Turkish Arabian foals.

**Table 2:** Least-squares means and their standard errors for body measurement at birth in Turkish Arabian foals.

Factors	n	BW ( kg )	WH ( cm )	CC ( cm )	CBC ( cm )
μ	2624	45.99 ± 0.12	97.55 ± 0.12	81.11 ± 0.10	11.54 ± 0.02
Farm		^∗∗^	^∗∗^	^∗∗^	^∗∗^
Anadolu Tİ	1583	45.09 ± 0.10^c^	96.55 ± 0.10^c^	79.81 ± 0.08^c^	10.54 ± 0.02^c^
Karacabey Tİ	541	45.95 ± 0.21^b^	98.40 ± 0.21^a^	81.47 ± 0.17^b^	11.53 ± 0.03^b^
Sultansuyu Tİ	500	46.94 ± 0.20^a^	97.71 ± 0.21^b^	82.06 ± 0.16^a^	12.55 ± 0.03^a^
Birth year		^∗∗^	^∗∗^	^∗∗^	^∗∗^
1987	46	46.08 ± 0.55^a,b,c,d,e,f^	96.12 ± 0.56^d,e,f,g,h^	80.59 ± 0.44^c,d,e,f,g,h^	11.76 ± 0.08^b,c,d^
1988	33	45.56 ± 0.64^a,b,c,d,e,f^	96.82 ± 0.66^b,c,d,e,f,g,h^	81.19 ± 0.52^b,c,d,e,f,g,h^	11.76 ± 0.09^b,c,d^
1989	73	45.89 ± 0.43^b,c,d,e,f^	95.76 ± 0.44^f,g,h^	79.34 ± 0.35^h^	11.43 ± 0.06^d^
1990	70	47.45 ± 0.45^a,b^	99.29 ± 0.46^a,b^	81.38 ± 0.37^b,c,d,e,f^	11.74 ± 0.07^b,c,d^
1991	74	47.40 ± 0.44^a,b^	100.34 ± 0.45^a^	82.13 ± 0.36^a,b,c^	12.24 ± 0.06^a^
1992	75	46.95 ± 0.44^a,b,c^	99.88 ± 0.45^a^	82.32 ± 0.35^a,b,c^	11.87 ± 0.06^b^
1993	76	45.98 ± 0.44^b,c,d,e^	98.39 ± 0.45^a,b,c,d,e^	81.58 ± 0.35^b,c,d,e^	11.71 ± 0.06^b,c,d^
1994	75	46.62 ± 0.44^a,b,c,d^	98.47 ± 0.45^a,b,c,d,e^	81.05 ± 0.36^b,c,d,e,f,g,h^	11.64 ± 0.06^b,c,d^
1995	69	47.11 ± 0.45^a,b,c^	98.89 ± 0.47^a,b,c^	81.03 ± 0.37^b,c,d,e,f,g,h^	11.74 ± 0.07^b,c,d^
1996	69	46.69 ± 0.45^a,b,c,d^	98.61 ± 0.46^a,b,c,d^	80.69 ± 0.37^c,d,e,f,g,h^	11.77 ± 0.07^b,c^
1997	78	46.05 ± 0.43^b,c,d,e^	98.79 ± 0.44^a,b,c^	79.92 ± 0.35^e,f,g,h^	11.63 ± 0.06^b,c,d^
1998	75	46.88 ± 0.44^a,b,c^	98.44 ± 0.45^a,b,c,d,e^	80.17 ± 0.35^d,e,f,g,h^	11.66 ± 0.06^b,c,d^
1999	145	47.93 ± 0.31^a^	97.13 ± 0.32^c,d,e,f^	82.45 ± 0.25^a,b^	11.52 ± 0.05^c,d^
2000	143	46.78 ± 0.31^a,b,c^	97.33 ± 0.32^b,c,d,e,f^	83.60 ± 0.25^a^	11.53 ± 0.05^c,d^
2001	239	44.39 ± 0.24^e,f^	96.74 ± 0.24^e,f,g^	81.07 ± 0.19^c,d,e^	11.57 ± 0.03^c,d^
2002	287	44.54 ± 0.23^e,f^	95.69 ± 0.22^g,h^	80.04 ± 0.18^f,g,h^	11.55 ± 0.03^c,d^
2003	292	44.97 ± 0.22^d,e,f^	98.68 ± 0.22^a,b^	79.94 ± 0.18^g,h^	11.50 ± 0.03^d^
2004	253	45.51 ± 0.23^c,d,e^	98.03 ± 0.24^b,c,d^	81.17 ± 0.19^c,d,e^	11.17 ± 0.03^e^
2005	174	44.31 ± 0.29^e,f^	95.11 ± 0.29^h^	80.97 ± 0.23^c,d,e,f,g^	10.97 ± 0.04^f^
2006	131	44.09 ± 0.33^f^	94.79 ± 0.34^h^	81.50 ± 0.27^b,c,d^	10.72 ± 0.05^g^
2007	147	44.70 ± 0.31^e,f^	95.31 ± 0.32^h^	81.23 ± 0.25^b,c,d,e^	10.85 ± 0.05^f,g^
Birth month		^∗∗^	^∗∗^	–	^∗^
1	438	45.13 ± 0.19^b^	97.00 ± 0.20^b^	81.18 ± 0.16	11.46 ± 0.03^b^
2	794	46.45 ± 0.16^a^	97.83 ± 0.16^a^	80.99 ± 0.13	11.56 ± 0.02^a^
3	708	46.39 ± 0.16^a^	97.85 ± 0.17^a^	81.19 ± 0.13	11.56 ± 0.02^a^
4	505	46.20 ± 0.19^a^	97.41 ± 0.20^a,b^	81.26 ± 0.16	11.57 ± 0.03^a^
5	179	45.81 ± 0.29^a,b^	97.68 ± 0.29^a,b^	80.94 ± 0.23	11.55 ± 0.04^a,b^
Sex		^∗∗^	^∗∗^	^∗^	^∗∗^
Male	1306	46.32 ± 0.14	97.91 ± 0.15	81.28 ± 0.12	11.60 ± 0.02
Female	1318	45.67 ± 0.14	97.20 ± 0.14	80.94 ± 0.11	11.48 ± 0.02
Dam age		^∗∗^	^∗∗^	^∗∗^	^∗∗^
≤ 7 year	804	44.33 ± 0.16^b^	96.29 ± 0.17^b^	79.93 ± 0.13^b^	11.35 ± 0.02b
≥ 8 and ≤ 12 year	1037	46.68 ± 0.15^a^	98.04 ± 0.15^a^	81.60 ± 0.12^a^	11.61 ± 0.02^a^
≥ 13	783	46.98 ± 0.16^a^	98.32 ± 0.17^a^	81.80 ± 0.13^a^	11.66 ± 0.02^a^

BW, birth weight; WH, wither height; CC, chest circumference; CBC, cannon-bone circumference. ^∗^ P < 0.01. ^∗∗^ P < 0.001. ^a, b, c, d, e, f, g, h^ The differences between subgroups in the same column are significant ( P < 0.05).

The least-squares means and ANOVA results for the body measurements at birth are shown in Table 2. The effects of farm, birth year, sex, and dam age on body measurements were statistically significant ( P < 0.001). BW, WH, and CBC were significantly ( P < 0.001) affected by the birth month. The overall mean values for BW, WH, CC, and CBC were 45.99 ± 0.12 kg , 97.55 ± 0.12, 81.11 ± 0.10, and 11.54 ± 0.02 cm , respectively.

**Table 3:** Variance components and genetic parameters for body measurements of Arabian foals at birth with LogL and AIC values for different models.

Traits	Model	$\sigma_{a}^{2}$	$\sigma_{\text{pe}}^{2}$	$\sigma_{m}^{2}$	$\sigma_{\text{am}}$	$\sigma_{e}^{2}$	$\sigma_{p}^{2}$	$h_{a}^{2}$	${\mathrm{pe}}^{2}$	$h_{m}^{2}$	$r_{\text{am}}$	$h_{T}^{2}$	LogL	AIC
BW	1	7.70 ± 0.95				5.98 ± 0.56	13.68 ± 0.54	0.56 ± 0.05				0.56 ± 0.05	- 4455.04	8914.08
	2	2.51 ± 0.76	2.31 ± 0.36			7.65 ± 0.47	12.46 ± 0.45	0.20 ± 0.06	0.19 ± 0.03			0.20 ± 0.06	- 4432.68	8871.36
	3	1.24 ± 0.52		3.11 ± 0.44		8.39 ± 0.39	12.75 ± 0.47	0.10 ± 0.04		0.24 ± 0.03		0.22 ± 0.04	- 4422.294	**8850.588**
	4	1.15 ± 0.49		2.60 ± 0.57	0.58 ± 0.47	8.45 ± 0.38	12.78 ± 0.47	0.09 ± 0.04		0.20 ± 0.04	0.34 ± 0.31	0.26 ± 0.05	- 4421.734	8851.468
	5	1.24 ± 0.52	0.61 ± 0.45	2.40 ± 0.65		8.32 ± 0.39	12.56 ± 0.47	0.10 ± 0.04	0.05 ± 0.04	0.19 ± 0.05		0.19 ± 0.04	- 4421.467	8850.934
	6	1.13 ± 0.49	0.58 ± 0.44	2.00 ± 0.69	0.50 ± 0.44	8.38 ± 0.38	12.59 ± 0.47	0.09 ± 0.04	0.05 ± 0.04	0.16 ± 0.05	0.33 ± 0.33	0.23 ± 0.05	- 4420.97	8851.94
WH	1	7.99 ± 0.96				5.88 ± 0.56	13.88 ± 0.55	0.58 ± 0.05				0.58 ± 0.05	- 4463.713	8931.426
	2	5.89 ± 1.03	1.35 ± 0.31			6.25 ± 0.58	13.48 ± 0.55	0.44 ± 0.06	0.10 ± 0.02			0.44 ± 0.06	- 4452.51	8911.02
	3	5.68 ± 1.08		1.48 ± 0.41		6.53 ± 0.60	13.69 ± 0.56	0.42 ± 0.07		0.11 ± 0.03		0.47 ± 0.06	- 4453.386	8912.772
	4	5.64 ± 1.27		1.46 ± 0.59	0.04 ± 0.72	6.55 ± 0.68	13.69 ± 0.56	0.41 ± 0.08		0.11 ± 0.04	0.02 ± 0.25	0.47 ± 0.06	- 4453.385	8914.77
	5	5.49 ± 1.06	0.89 ± 0.39	0.65 ± 0.47		6.45 ± 0.59	13.48 ± 0.55	0.41 ± 0.07	0.07 ± 0.03	0.05 ± 0.03		0.43 ± 0.06	- 4451.148	**8910.296**
	6	5.75 ± 1.30	0.92 ± 0.40	0.77 ± 0.58	- 0.27 ± 0.69	6.32 ± 0.70	13.49 ± 0.55	0.43 ± 0.09	0.07 ± 0.03	0.06 ± 0.04	- 0.13 ± 0.30	0.42 ± 0.07	- 4451.086	8912.172
CC	1	2.01 ± 0.43				5.30 ± 0.31	7.31 ± 0.25	0.27 ± 0.05				0.27 ± 0.05	- 3799.123	7602.246
	2	0.68 ± 0.28	0.81 ± 0.17			5.57 ± 0.24	7.06 ± 0.22	0.10 ± 0.04	0.11 ± 0.02			0.10 ± 0.04	- 3786.427	7578.854
	3	0.46 ± 0.24		0.92 ± 0.19		5.76 ± 0.23	7.15 ± 0.23	0.07 ± 0.03		0.13 ± 0.03		0.13 ± 0.03	- 3782.062	7570.124
	4	0.49 ± 0.27		0.98 ± 0.30	- 0.07 ± 0.26	5.75 ± 0.24	7.15 ± 0.23	0.07 ± 0.04		0.14 ± 0.04	- 0.10 ± 0.36	0.12 ± 0.04	- 3782.034	7572.068
	5	0.43 ± 0.23	0.33 ± 0.20	0.62 ± 0.24		5.70 ± 0.23	7.09 ± 0.22	0.06 ± 0.03	0.05 ± 0.03	0.09 ± 0.03		0.10 ± 0.03	- 3780.722	**7569.444**
	6	0.48 ± 0.27	0.35 ± 0.21	0.70 ± 0.32	- 0.11 ± 0.24	5.67 ± 0.24	7.09 ± 0.22	0.07 ± 0.04	0.05 ± 0.03	0.10 ± 0.04	- 0.19 ± 0.37	0.09 ± 0.04	- 3780.624	7571.248
CBC	1	0.15 ± 0.02				0.15 ± 0.01	0.30 ± 0.01	0.51 ± 0.05				0.51 ± 0.05	441.816	- 879.632
	2	0.10 ± 0.02	0.03 ± 0.01			0.16 ± 0.01	0.29 ± 0.01	0.34 ± 0.06	0.11 ± 0.02			0.34 ± 0.06	452.138	- 898.276
	3	0.09 ± 0.02		0.04 ± 0.01		0.17 ± 0.01	0.29 ± 0.01	0.30 ± 0.07		0.13 ± 0.03		0.37 ± 0.06	455.29	-904.58
	4	0.10 ± 0.03		0.05 ± 0.01	- 0.01 ± 0.02	0.16 ± 0.01	0.29 ± 0.01	0.34 ± 0.09		0.16 ± 0.05	- 0.18 ± 0.21	0.35 ± 0.06	455.52	- 903.04
	5	0.09 ± 0.02	0.01 ± 0.01	0.03 ± 0.01		0.17 ± 0.01	0.29 ± 0.01	0.30 ± 0.06	0.04 ± 0.03	0.10 ± 0.04		0.35 ± 0.06	455.751	- 903.502
	6	0.10 ± 0.03	0.01 ± 0.01	0.04 ± 0.02	- 0.01 ± 0.02	0.16 ± 0.02	0.29 ± 0.01	0.34 ± 0.09	0.04 ± 0.03	0.12 ± 0.06	- 0.22 ± 0.23	0.34 ± 0.06	456.05	- 902.1

Abbreviations: $\sigma_{a}^{2}$ , additive genetic variance; $\sigma_{\text{pe}}^{2}$ , maternal permanent environmental variance; $\sigma_{m}^{2}$ , maternal genetic variance; $\sigma_{\text{am}}$ , covariance between additive and maternal genetic effect; $\sigma_{e}^{2}$ , residual variance; $\sigma_{P}^{2}$ , phenotypic variance; $h_{a}^{2}$ , additive heritability; ${\text{pe}}^{2}$ , ratio of maternal permanent environmental variance to phenotypic variance; $h_{m}^{2}$ , maternal heritability; $r_{\text{am}}$ , correlation between additive and maternal genetic effect; $h_{T}^{2}$ , total heritability; BW, birth weight; WH, wither height; CC, chest circumference; CBC, cannon-bone circumference. The best-fit model according to AIC is shown in bold type.

Table 3 presents a comprehensive summary of covariance components, genetic parameters, and log-likelihood (LogL) and Akaike information criterion (AIC) values for each model applied to analyze body measurements at birth in Turkish Arabian foals. The model that best explained the sources of variation in the traits was determined based on Akaike's information criterion (AIC) values. The best-fit model for each trait was chosen to yield the lowest AIC values. According to the AIC values, the best-fit models explaining the variation in BW, WH, CC, and CBC were models 3, 5, 5, and 3, respectively. The direct additive heritabilities for BW and CC were low (0.10 ± 0.04 and 0.06 ± 0.03), while estimates for WH and CBC were found to be moderate (0.41 ± 0.07 and 0.30 ± 0.07). The maternal heritability values for the corresponding traits were 0.24 ± 0.03, 0.05 ± 0.03, 0.09 ± 0.03, and 0.13 ± 0.03, respectively. Permanent environmental effects emerged in WH (0.07 ± 0.03) and CC (0.05 ± 0.03), since Model 5 was the best fit. Although the models with covariance between offspring and dams did not yield the lowest AIC in the analyses, genetic correlations between direct and maternal genetic effects ranged from - 0.22 to 0.34. The total heritabilities were 0.22 ± 0.04, 0.43 ± 0.06, 0.10 ± 0.03, and 0.37 ± 0.06, respectively.

**Table 4:** Genetic (above diagonal) and phenotypic (below diagonal) correlations among body measurement traits.

	BW	WH	CC	CBC
BW	–	0.824 ± 0.083	0.736 ± 0.165	0.924 ± 0.075
WH	0.688 ± 0.013	–	0.340 ± 0.225	0.829 ± 0.073
CC	0.626 ± 0.014	0.522 ± 0.017	–	0.458 ± 0.229
CBC	0.626 ± 0.014	0.618 ± 0.015	0.435 ± 0.018	–

Abbreviations: BW, birth weight; WH, wither height; CC, chest circumference; CBC, cannon-bone circumference.

Table 4 displays the genetic and phenotypic correlations between the traits derived from multivariate analyses. Positive genetic correlations were obtained using multivariate analysis. Very strong positive genetic correlations (0.824 ± 0.083 and 0.924 ± 0.075) were observed between BW and WH and CBC, respectively. CC is strongly and positively correlated with only BW, whereas correlations between CC and WH, and CBC were weak to moderate (0.340 ± 0.225 and 0.458 ± 0.229). Body measurements at birth showed phenotypic correlations ranging from moderate to strongly high (0.435 ± 0.018 to 0.688 ± 0.013).

**Figure 1:**
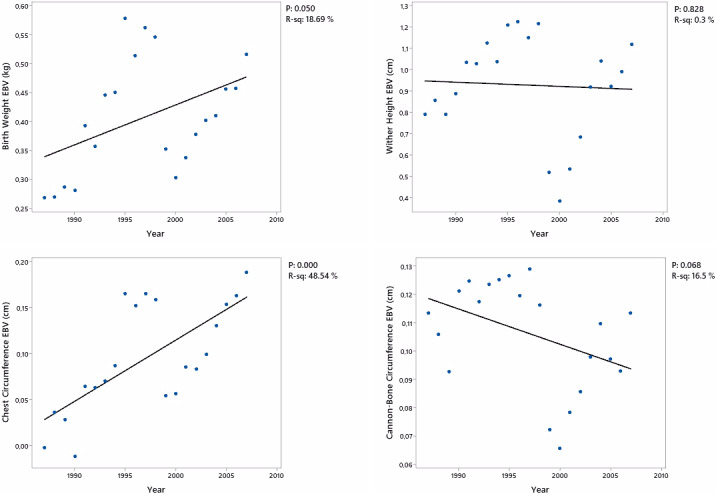
Genetic trend of estimated breeding values (EBVs) for body measurement traits at birth in the Arabian horse population.

The genetic trends for body measurements of foals are shown in Fig. 1, with corresponding P and $R^{2}$ values. Although the trends for BW and CC were significant ( P<0.05 ), there was no significant trend for WH. The CBC showed a decreasing trend with a tendency level ( P < 0.10).

## 4 Discussion

BW was significantly affected ( P < 0.001) by farm operation, and the highest BW was obtained from Sultansuyu (46.94 kg ), followed by Karacabey (45.95 kg ) and Anadolu (45.09 kg ). The diversity in management practices among various farm operations may have contributed to this situation. The birth year and month also had significant ( P < 0.001) effects on BW. Colts (46.32 kg ) were heavier than fillies (45.67 kg ). The age of mares had a significant ( P < 0.001) effect, and foals born from mares older than 8 years of age were heavier than the others. This may indicate the better maternal environment provided by multiparous mares to foals than that provided by primiparous mares. The mean BW (45.99 kg ) was in line with that reported by Kaygısız et al. (2011) and Altınel and Küçük (1992) in purebred Arabian horses. These values were also lower than the reports of de Castro et al. (2021) and Dall'Anese et al. (2023) for thoroughbred foals and higher than the results of Koç and Altınel (1992) for Arabian and Huricha et al. (2022) for Hokkaido native foals. The wither height (97.55 cm ) was found to be higher than that reported by Altınel and Küçük (1992), Koç and Altınel (1992), Kaygısız et al. (2011), Çilek (2012), and Filho et al. (2014) for purebred Arabian horses and Huricha et al. (2022) for native Hokkaido foals. However, de Castro et al. (2021) and Dall'Anese et al. (2023) reported that higher values ranged from 102.2 to 103.6 cm for this trait in thoroughbred foals. The CC (81.11 cm) was close to the values reported by Filho et al. (2014) and Altınel et al. (1992) for purebred Arabian horses. While this was lower than the finding of Kaygısız et al. (2011), it was greater than the values obtained by Çilek (2012) and Koç and Altınel (1992) for Arabian and Huricha et al. (2022) for Hokkaido breeds. CBC (11.54 cm ) was higher than that reported for Arabian and Hokkaido horses (Çilek, 2012; Filho et al., 2014; Huricha et al., 2022) and similar to the findings of Kaygısız et al. (2011). These variations can be attributed to factors such as horse breed, the statistical model employed, and management practices.

This investigation unveiled the covariance components, incorporating additive genetic, maternal genetic, and maternal permanent effects and the covariance between offspring and dams, employing various models to scrutinize body measurements at birth in Arabian foals.

In this study, the estimated additive direct heritabilities for BW, WH, CC, and CBC were 0.10 ± 0.04, 0.41 ± 0.07, 0.06 ± 0.03, and 0.30 ± 0.07, respectively, with the direct heritability of BW (0.10 ± 0.04) being low and consistent with the results of Kaygısız et al. (2011), indicating limited genetic influence and the significant role of environmental factors in this trait. The moderate heritability estimate (0.41 ± 0.07) for WH aligns closely with previous findings reported by Bakhtiari and Heshmat (2009) and Mehta et al. (2021), reflecting a consistent genetic influence on this trait across studies. However, the literature presents a wide range of heritability estimates for WH, underscoring its variability across different breeds and ages. Studies by Doğan et al. (2002), Pretorius et al. (2004), Dario et al. (2006), Antalyalı(2008), Kaps et al. (2011), and Poyato-Bonilla et al. (2021) reported lower estimates ranging between 0.19 and 0.30, while Kaygısız et al. (2011) found a notably lower heritability of 0.10 for Arabian foals. Conversely, higher heritability estimates for WH, ranging from 0.47 to 0.89, have been reported in studies by Seidlitz et al. (1991), Molina et al. (1999), Druml et al. (2008), Gharahveysi et al. (2008), de Almeida Prado and da Mota (2008), Viklund et al. (2008), Schroderus and Ojala (2010), Gücüyener Hacan and Akçapınar (2011), Çilek (2012), Tamioso et al. (2012), Duru et al. (2017), Giontella et al. (2020), and Müller et al. (2021). This broad range of estimates underscores the complex interplay of genetic and environmental factors in determining WH across different horse breeds and developmental stages. Such variability emphasizes the necessity of considering breed-specific genetic backgrounds and environmental influences when assessing the heritability of WH in foals. Maternal heritabilities from the six models for WH (ranged between 0.05 and 0.11) were lower than the previously estimated value (0.14) in Arabian horses at the age of 1 and 2 years by Duru et al. (2017). This may be attributed to the fact that maternal genetic effects remained as the foals grew because of suckling milk from the dam and the maternal environment. The permanent environmental effect was found to be 0.07 in this trait, and this presumably would lead to a smaller maternal heritability in Model 5. The heritability estimate (0.06 ± 0.03) for CC in the current study was very low, and this value shows similarity with the range of 0.03 to 0.08 from earlier reports (Kaps et al., 2011; Kaygısız et al., 2011; Çilek, 2012; Müller et al., 2021). On the other hand, markedly higher estimates (0.21–0.66) were also determined (Seidlitz et al., 1991; Molina et al., 1999; Doğan et al., 2002; Dario et al., 2006; Sadek et al., 2006; Antalyalı, 2008; Druml et al., 2008; Gharahveysi et al., 2008; de Almeida Prado and da Mota, 2008; Bakhtiari and Heshmat, 2009; Gücüyener Hacan and Akçapınar, 2011; Duru et al., 2017; Giontella et al., 2020; Mehta et al., 2021; Poyato-Bonilla et al., 2021). The relatively low additive heritability identified for this trait in the study may stem from the significant influence exerted by mares on the total variance, encompassing both genetic and permanent factors. The direct additive heritability estimate (0.30 ± 0.07) for CBC was in concordance with the values between 0.22 and 0.30 obtained in different studies (Doğan et al., 2002; Bakhtiari and Heshmat, 2009; Çilek, 2012). This value was higher than the range of 0.05 to 0.17 (Antalyalı, 2008; Gharahveysi et al., 2008; Kaygısız et al., 2011; Filho et al., 2014; Müller et al., 2021; Poyato-Bonilla et al., 2021) for Arabian, Campolina, Mangalarga Marchador, Quarter, Iranian Arab, Criollo, and Pura Raza Española breeds. Conversely, studies (Seidlitz et al., 1991; Molina et al., 1999; Pretorius et al., 2004; Dario et al., 2006; Druml et al., 2008; de Almeida Prado and da Mota, 2008; Gücüyener Hacan and Akçapınar, 2011; Duru et al., 2017; Giontella et al., 2020) conducted in different breeds revealed higher estimated heritabilities ranging from 0.35 to 0.57 for CBC. The differences may have originated from factors such as the number of animals, breed, and ages. Maternal genetics and permanent environmental variations showed that these factors had some but low effects on fetal development. The uterine environment provided by mares may affect the size of foals in birth. Significant yet low direct heritabilities suggest that genetic progress could be attained by precisely managing environmental factors. Huricha et al. (2022) aimed to estimate direct and maternal heritability of body measurements at birth, but they found overestimated values. The covariance between offspring and dams indicates that the improvements in one effect will lead to recession in the other (Southwood and Kennedy, 1990). The correlations between offspring and dams for BW, WH, CC, and CBC from Model 6 were 0.33, - 0.13, - 0.19, and - 0.22, respectively. Positive additive–maternal correlation observed in BW (0.33) showed that the weight is affected not only by the additive genetics of the animal but also by maternal genetics. Supporting results were presented by Dodenhoff et al. (1998), who found 0.25 positive $r_{\text{am}}$ for the birth weight of Hereford cattle. Torzyński et al. (2005) reported that low correlation between offspring and dams ranged from - 0.20 to 0.01 for WH, CC, and CBC in half-bred horses. Negative additive–maternal genetic correlations were also found in several species such as sheep, cattle, and sows (Southwood and Kennedy, 1990; Aksoy et al., 2016; Koçak et al., 2024; Illa et al., 2024). In general, negative $r_{\text{am}}$ would be biologically impossible, and the probable reason for negative $r_{\text{am}}$ was reported to be the poor environment or data structure (Meyer, 1992; Maniatis and Pollott, 2003). Although the correlations between offspring and dams were low, not including the additive–maternal covariance may lead to under- or overestimation of the direct heritability. Our study represents the first attempt to examine the variances of additive genetic, maternal genetic, and maternal permanent environmental effects, along with the covariance between offspring and dams. We employed six different models to identify the most suitable one for elucidating the variation in body measurements at birth among purebred Arabian horses in Türkiye.

The phenotypic correlation (0.688 ± 0.013) between BW and WH at birth was close to the value (0.745) found by Kaygısız et al. (2011) for Arabian horses. Phenotypic correlations of BW–CC (0.626 ± 0.014) and BW–CBC (0.626 ± 0.014) were higher than those presented by Kaygısız et al. (2011). Phenotypic and genetic correlations between WH and CC were lower than the range of 0.53 to 1.00 in the literature (Doğan et al., 2002; Falcao et al., 2002; Sadek, 2006; Kaygısız et al., 2011). Giontella et al. (2020) reported greater genetic correlation (0.71) between WH and CC in Sardinian Anglo Arab horse than in the breeds detected in our research. However, lower correlations (0.09 and 0.458) were reported for different horse breeds (Falcao et al., 2002; Barzev et al., 2003; Baban et al., 2009). In the current study, correlations, both phenotypic and genetic, for WH and CBC were moderately high, surpassing the findings of various researchers (Falcao et al., 2002; Barzev et al., 2003; Sadek, 2006; Kaygısız et al., 2011). On the other hand, these correlations were not as high as those estimated by Baban et al. (2009) or as those reported by Doğan et al. (2002). Lower genetic correlation was found by Giontella et al. (2020) for Sardinian Anglo Arab horse. Strong genetic correlation in a favorable direction indicates that animals with thicker CBC have higher withers. Consistently, Sadek et al. (2006) found high genetic correlation (0.77) among these traits. Phenotypic and genetic correlations between CC and CBC were moderate and agree with the values of Barzev et al. (2003) and Baban et al. (2009) for Hanoverian and Lipizzaner breeds but lower than values in some research (Doğan et al., 2002; Falcao et al., 2002; Sadek et al., 2006; Kaygısız et al., 2011; Giontella et al., 2020).

The significant ( P < 0.05) trends for both BW and CC in foals indicated that the selection of performance could implicitly result in increases in some specific body measurements of foals. Conversely, WH did not show a statistically significant trend. The breed-specific criteria considered in body measurements for breeding may have hindered the emergence of a trend in wither height. The CBC showed a trend towards significance ( P < 0.10), indicating a gradual decrease. This trend indicates a modest decrease in CBC over time, although it did not reach statistical significance at conventional levels.

## 5 Conclusion

The results highlighted that the selection for the performance of horses significantly impacts the genetic variation of foals' body size at birth. Notably, BW and CC exhibited significant trends under selection, while WH remained stable. Heritability estimates for these measurements were low to moderate, indicating the substantial role of both genetic and environmental factors. Effective breeding programs should consider these heritabilities and the associated genetic and phenotypic correlations. Breeders could enhance the performance of racehorses more effectively by adopting a holistic approach accounting for genetic and environmental factors.

## Data Availability

The data used in this study are available from Özlem Hacan (ogucuyener@gmail.com) upon reasonable request.
